# Effect of pelleted vs. ground starter with or without hay on preweaned dairy calves

**DOI:** 10.1371/journal.pone.0234610

**Published:** 2020-07-09

**Authors:** Aloma Eiterer Leão, Sandra Gesteira Coelho, Rafael Alves Azevedo, Mariana Magalhães Campos, Fernanda Samarini Machado, Juliana Guimarães Laguna, Alexandre Lima Ferreira, Luiz Gustavo Ribeiro Pereira, Thierry Ribeiro Tomich, Suely de Fátima Costa, Marco Antônio Machado, Daniele Ribeiro de Lima Reis

**Affiliations:** 1 Department of Animal Science, Veterinary School, Federal University of Minas Gerais, Belo Horizonte, Minas Gerais, Brazil; 2 EMBRAPA - Dairy Cattle Research Center, Juiz de Fora, Minas Gerais, Brazil; 3 Department of Animal Sciences, Michigan State University, East Lansing, Michigan, United States of America; 4 Department of Veterinary Medicine, Federal University of Lavras, Lavras, Minas Gerais, Brazil; Universidade Federal de Viçosa, BRAZIL

## Abstract

The objective of this study was to evaluate the effect of the physical form of starter and inclusion of hay in the diet of preweaning dairy calves on performance, digestibility, ruminal development, and mRNA expression of genes involved in ruminal metabolism. Holstein × Gyr crossbred male calves (n = 38 1day old) were assigned to 3 treatments for 9 weeks: Control (n = 13; pellet starter with 4 mm diameter and 18 mm length and 4% steam-flaked corn), Ground (n = 12; same starter of the control but ground pass through a 4.0 mm sieve), or Ground plus 5% chopped Tifton hay GH (n = 13). All calves were fed 4 L/d of whole milk up to 63 d of age and were abruptly weaned at 64 d of age. Water and diets were offered *ad libitum*. Samples of ruminal contents were obtained from all animals at 30, 45, and 60 d of age to evaluate pH, ammonia nitrogen, and volatile fatty acids (VFA). At 55 d of age, an apparent digestibility assay was performed using 18 animals (n = 6/ treatment). At 65 d of age, the 18 animals were euthanized to evaluate the development of the digestive tract. The physical form of starter and the dietary inclusion of hay did not influence starter intake (Control 326 g/d, Ground 314 g/d and GH 365 g/d), daily weight gain (Control 541g/d, Ground 531g/d and GH 606g/d), feed efficiency, apparent nutrient digestibility, energy partitioning, nitrogen balance, ruminal pH, ammonia nitrogen concentration, VFA, the development of the digestive tract and the mRNA expression of genes involved in AGV metabolism.

## Introduction

According to the NRC 2001 [1], besides being palatable, calf starter must be made of good quality ingredients to ensure adequate supply of protein, energy, fiber, vitamins, and minerals while also having a coarse texture. Coarse-textured starter is indicated to stimulate ruminal motility, musculature development, and epithelial health [2], and the use of readily fermentable carbohydrates is indicated to stimulate the development of the ruminal epithelium [3, 4].

The consumption of readily fermentable carbohydrates increases the concentrations of propionate and butyrate in ruminal fluid, which are absorbed and metabolized by the ruminal epithelium. Volatile fatty acids (VFA), specially butyrate, can stimulate the ruminal papillae development [5, 4, 6].

According to [7], feedstuffs processing can modify the physical and chemical form of food, altering ruminal fermentation and digestibility. Therefore, processing can affect intake and the physical and metabolic development of the digestive tract [4]. However, the lack of synergism between the fermentation of carbohydrates and the absorption of the end products of fermentation can decrease ruminal pH, leading to abnormal epithelial development [8], in addition to reduced absorption of VFA [9].

The supply of finely ground starter can be economically advantageous compared to the textured starter (due to the use of extruded soya, steam-flaked corn among other processed grains). However, the finely ground concentrate can also present a fast fermentation rate and a drop in rumen pH. An alternative to avoid abnormal development of rumen epithelium in this situation would be the inclusion of hay in the calf diet [10, 11].

To better understand the mechanisms responsible for variations in intake and performance in response to physical changes in diets, some researchers have associated molecular techniques and dietary nutrients analysis, objectifying to quantify and understand what genes are involved in the ruminal metabolism of VFA, optimize papilla growth, and ruminal metabolism [12, 13, 14]. According to [15], providing rumen-degradable starch rather than rumen-degradable fiber to heifers, increased the mRNA expression of propionyl-CoA carboxylase A (*PPCA*) in the ruminal epithelium, the ruminal surface area and papillae length all being higher. Calves that consumed a greater amount of starter had more VFA concentration in the rumen, up-regulated genes involved in the ruminal VFA transporter, cell proliferation, ketogenic and propionate metabolism, and down-regulated genes involved in the fatty acid oxidation [16, 13].

The objectives of this study were to evaluate the effect of the physical form of starter and hay provision in the diet of dairy calves during the preweaning period on growth, nutrient digestibility, energy partitioning, nitrogen balance, rumen development and level of mRNA expression of genes involved in ruminal metabolism. Our hypothesis was that a pellet starter with 4% steam-flaked corn and ground starter plus 5% chopped can improve ruminal development and alters the mRNA expression of genes involved in rumen metabolism.

## Materials and methods

This study was approved by the Ethics Committee of Embrapa Dairy Cattle, Brazil (protocol number 26/2015). The experiment was conducted at the Embrapa Dairy Cattle Experimental Farm, located in Coronel Pacheco, Minas Gerais, Brazil.

### Animals, housing, and treatments

Holstein × Gyr crossbred male calves (n = 38, birth body weight (BW) = 33.8 ± 4.3 kg, mean ± SD) with genetic composition 5/8 Holstein were used. Calves were born between September to December 2015. At birth, calves were removed from their dams, weighed and had their umbilical cord immersed in iodine solution (7%). Colostrum, 10% of birth weight (Brix > 22%), was fed within 6–8 h after birth. Blood samples were collected via jugular vein puncture within 48 h after birth. Samples were centrifuged at 800.6 × *g* for 10 min to measure total serum protein using a digital refractometer (Misco DD-3 Palm Abbe Digital, Solon, Ohio, USA). Calves were reared in individual shelters over sand (1.25 x 1.75 m), which were allocated in a barn with open sides. At 8 d of age, preventive oral treatment against coccidiosis (Baycox Ruminants, Bayer, Leverkusen, Germany) was performed, at 3 mL/10 kg of BW.

Until to 3 d of age, calves were fed 4 L/d of transition milk (days 2 and 3 post-partum) divided into two equal meals offered at 0800 and 1600 h. At 4 d, calves were assigned to 3 treatments, maintaining the same distribution of birth month, birth BW, total serum protein, and genetic composition. The treatments were: Control (n = 13) pellet starter with 4% steam-flaked corn (Soylac Rumen 20% CP, Total Alimentos, Três Corações, Minas Gerais, Brazil) (Table 1), Ground (n = 12) same starter of the control but ground pass through a 4.0 mm sieve), or GH (n = 13) same starter of the control but ground pass through a 4.0 mm sieve plus 5% chopped Tifton (*Cynodon* spp.) hay. The particle size for each starter, after pass a 4,0 mm diameter mesh screen, was quantified using GRANUTEST^®^ (Telastem, São Paulo, Brazil). Particle size of control, % retained for sieves of 4,0; 2,0; 1,19; 0,6; 0,3; 0,15; 0,04 mm was respectively 78,28; 10,29; 6,00; 2,55; 1,56; 0,66; and 0,66; and for starter of treatments Ground and GH was 0,58; 2,24; 18,51; 32,15; 30,10; 12,57; and 3,85. For treatment GH, hay was offered separately from starter at 5% of the amount of starter supplied. The hay was chopped using a stationary chopper (Menxon Charger 35.0 G, Cajuru, São Paulo, Brazil), and the particle distribution was determined using the NASCO’s Penn State Particle Separator (Nasco, Fort Atkinson, WI—[17]); as follows it was composed of 67.1% long particles (>19 mm), 13.3% medium particles (between 8 and 20 mm), and 19.6% small particles (<8 mm).

**Table 1:** Nutrient composition (DM basis, % unless otherwise noted) of whole milk, starters, and hay.

Item	Whole milk	Starter^1^	Hay
**DM**	12.6	85.8	84.4
**CP**	23.5	21.6	10.6
**Ether extract**	33.6	2.7	1.8
**Ash**	7.4	12.4	7.7
**Lactose**^2^	35.4	-	-
**NDF**	-	21.9	72.9
**ADF**	-	10.0	33.4

^1^As fed basis. Basic ingredients of minerals and vitamins in starter: calcitic limestone, sodium chloride, corn gluten meal 60%, soybean meal, wheat bran, dicalcium phosphate, calcium iodate, molasses, steam-flacked corn, ground shelled corn, toasted full-fat soybeans, vitamin A, vitamin D3, vitamin E, cobalt sulphate, copper sulphate, ferrous sulphate, manganese sulphate, sodium and calcium aluminosilicate, zinc sulphate, probiotic additive (*Saccharomyces cerevisiae*) and monensin sodium.

^2^Lactose % of treatments = 100 –CP%—EE%—Ash% - 2 [18].

From 4 to 63 d of age, the total volume of whole milk (4 L/d) was divided into two equal meals (0800 and 1600 h) and provided to calves in buckets. Calves were weaned at 64 d of age. Starter and water were offered *ad libitum* throughout the experimental period. The amount of starter provided was enough to result in approximately 20% orts.

### Intake and growth

Calves’ growth, body measurements, and intake were monitored between 4 and 63 d of age. Intakes of whole milk, starter, hay, and water were measured once a day, by subtracting refusals from the amounts provided. Water intake was measured using a portable balance (WH-A04; WeiHeng, Yongkang, China).

The BW and body measurements were obtained once a week before the morning feeding in a flat location, allowing animals to remain with their limbs well set. The height at withers and hip were evaluated using a hipometer (Walmur, Porto Alegre, Brazil), and hip width was assessed with a measuring tape (cm scale) (Bovitec, São Paulo, Brazil). Feed efficiency was calculated using the ratio between ADG and total DMI (includes milk DM) [19].

### Apparent nutrient digestibility, energy partitioning and nitrogen balance

Between 55 and 60 d of age, 6 animals per treatment were submitted to a total tract nutrient digestibility trial with 5 d total feces and 1 d urine collection in metabolic cages (dimensions of 1.50 m x 0.80 m; Intergado Ltda., Contagem, Brazil). During these 5 days, the fecal excretions of the animal were collected on the floor of the metabolic cage, and the total of feces was weighed and homogenized at the end of every 24 h. Equivalent quantities of the daily sub-samples were composited to one sample per animal and were stored at -20°C for further analysis. Urine collections were performed for 24 h [20]. The urine tray was drained into 5 L containers held in ice-filled polystyrene thermal boxes [21]. The volume, weight, and density of the urine from each animal were measured, and a sample of 50 mL was taken after it was filtered in cheesecloth and then stored at -20°C for gross energy and nitrogen analysis.

For the energy balance, gross energy intake (GEI) was calculated by the difference between the gross energy content (GE) of each feed offered (milk, starter, hay) and those obtained in the respective orts. Digestible energy intake (DEI) was calculated by the difference between GEI and fecal energy excretion. Metabolizable energy intake (MEI) was derived as the difference between DEI and urine energy. The percentages of consumed energy lost as feces and urine (DE/GE) and the relationships between ME/GE (metabolizability) and ME/DE, as energy efficiency indexes, were also calculated. Nitrogen balance, or nitrogen retained, was calculated according to the following equation: N retained (g/d) = N ingested—(fecal N + urine N).

### Nutrient analysis

During the entire experiment, milk samples were collected twice a day (morning and afternoon) and analyzed for total solids, crude protein (CP), lactose, and fat content using spectrophotometry (Bentley 2000; Bentley Instruments Inc., Chaska, MN). Samples of starter and hay were collected 3 d per week, composited biweekly and stored at −20°C until analysis. All samples of feed (experiment and digestibility assay) and feces were oven-dried at 55°C for 72 h, ground through a 1 mm mesh diameter sieve in a Wiley type mill (model 3, Arthur H. Thomas Co., Philadelphia, PA), and analyzed.

Samples of the starter and hay were analyzed for dry matter (DM) (Method 934.01), CP (Method 988.05), ether extract (EE) (Method 920.39), and ash (Method 942.05), according to the [22]. Neutral detergent fiber (NDF) and acid detergent fiber (ADF) were determined according to the method proposed by [23], with amylase, adapted to the conditions of the Ankom 220 apparatus, without the use of sodium sulphite and corrected for residual ash [24], ADF [25], and EE [25] (Method 920.39). For nitrogen compounds, the correction of NDF and ADF as well as the estimation of insoluble nitrogen in FDN and FDA, followed the recommendations of [26]. Gross energy (GE) was determined using an adiabatic calorimeter (IKA—C5000, IKA^®^ Works, Staufen, Germany). All analysis were performed at the Laboratory of Food Analysis of Embrapa Dairy Cattle.

### Ruminal pH, ammonia, and VFA

Ruminal fluid samples were obtained from 6 animals per treatment at 30, 45, and 60 d of age. Samples of approximately 50 mL were collected using a stomach tube technique [27, 28] 5 h after morning feeding. A 5-mL sample of ruminal content was added to 1 mL of 20% metaphosphoric acid for VFA analysis and another sample to 5 mL was used for ammonia concentration analysis. Ruminal pH fluid was measured immediately after collection using a portable pH meter (DM-2-Digimed, São Paulo, São Paulo, Brazil). Contents were filtered through four layers of cheesecloth to separate the liquid and solid fractions. All samples were stored in plastic tubes at -20°C for further analysis. The ruminal NH_3_N concentration was quantified after distillation of Kjeldahl with magnesium oxide and calcium chloride according to Method 920.03 [22]. Concentration of VFA was determined by HPLC in a Dionex Ultimate 3000 Dual Detector HPLC (Dionex Corporation, Sunnyvale, CA) coupled to a hodex RI-101 refractive index detector (ECOM Ltd., Prague, Czech Republic) maintained at 40°C using a Phenomenex Rezex ROA ion exchange column (Phenomenex, Torrance, CA), 300 × 7.8 mm, maintained at 45°C. Mobile phase was prepared with 5 mmol/L H2SO4, and the flow rate was 0.7 mL/min. Samples of 2 mL were defrosted at room temperature (22–25°C) and centrifuged (1,800 × *g*, 10 min). Cell-free supernatants were treated as described by [29]. The following acids were used to calibrate the standard curve: acetic, succinic, formic, lactic, propionic, valeric, isovaleric, isobutyric, and butyric. These acids were prepared to a final concentration of 10 mmol/L except isovaleric acid (5 mmol/L) and acetic acid (20 mmol/L).

### Slaughter, Rumen and Omasum measurements

At 65 d of age, seven calves from each treatment were euthanized after being deprived of liquid diet for 12 h. Pre-anesthetic medication with 2% xylazine hydrochloride (30 to 40 mg per 100 kg BW) was administered intravenously. After 20 minutes, 5% thiopental sodium (5 mg/kg BW) was directly administered into the foramen magnum, located in the occipital bone of the skull. Once the cardiorespiratory arrest was verified, bleeding was carried out by an incision made in the jugular furrow at the base of the neck.

After slaughter, the abdominal cavity was immediately opened, and each region of the gastrointestinal tract (reticulum-rumen, omasum, and abomasum) was isolated, tied off, and weighed. After samples were collected from the gastrointestinal tract, it was emptied, washed with running water (to remove contents), and weighed. Internal organs and viscera were also weighed. All variables were evaluated as a proportion of empty body weight (BW).

An area of 5 cm^2^ from the ventral ruminal sac and omasum laminae were fixed in formalin and processed for paraffin embedding. The paraffin blocks were sectioned using an Olympus CUT 4055 manual microtomes (Olympus, Tokyo, Japan) into 5 μm-thick serial sections. For morphometric papillae analysis, slides were stained with Hematoxylin-Eosin as described by [17]. Images were captured using a light microscope (CX31; Olympus) attached to a camera (OSIS SC30; Olympus) using Cell-B software (Olympus). Morphometric analysis were performed using AxioVision 4.8.2–06/2010 software (Carl Zeiss Images Systems, Jena, Germany). The measurements taken were height of ruminal and omasal papillae [18]. To determine mitotic index, 2,000 cells from the basal layer of the rumen and omasum epithelium were counted, including those with nuclei presenting mitotic figures (using the light microscope, 400× magnification). The mitotic index was calculated as a ratio between the number of nuclei in division and the total number of nuclei [19].

### Tissue collection and RNA extraction

Tissue samples were collected from the anterior portion of the ventral sac of the rumen [13, 20, 33] at 65 d after birth. The rumen epithelium was manually separated from the underlying muscular layer by gloved hands and rinsed in tap water to remove residual feed particles. Samples of rumen epithelial tissue were stored in RNA*later* (Thermo Fisher Scientific, Waltham, Massachusetts, USA) and thereafter stored at -20°C. The rumen tissue (30mg) was macerated using 600 μL of RLT buffer and 6 μL β-Mercaptoethanol using the Tissueruptor equipment (Qiagen, Hilden, Germany), and total RNA was extracted using RNeasy Mini kit (Qiagen, Hilden, Germany) and treated with DNase using RNase-Free DNase Set kit (Qiagen, Hilden, Germany), according to the manufacture’s protocol. After that, the quantity and purity of RNA were determined by absorbance at 260 and 280 nm using the NanoDrop ND-1000 spectrophotometer (Thermo Fisher Scientific, Waltham, Massachusetts, USA). The total RNA integrity was evaluated using the 2100 Bioanalyzer (Agilent, Santa Clara, CA, USA).

### Real-time quantitative PCR

For reverse transcription, 6 μL of total RNA was added to 1 μL Oligo(dT)_20_ and 1 μL annealing buffer. Then, this mixture was incubated at 65°C for 5 min and kept on ice for 3 min. After that, 10 μL 2X First-Strand Reaction Mix and 2 μL of SuperScript^™^ III (Invitrogen^®^) were added to this mixture and then incubated at 25°C for 5–10 min, 50°C for 50 min, and 85°C for 5 min.

Primer sequences for the following target genes, peroxisome proliferator-activated receptor delta (*PPARD*), β-hydroxybutyrate dehydrogenase-1 (*BDH1*), 3-Hydroxymethylglutaryl-CoA synthase, isoform I (*HMGCS1*), 3-Hydroxymethylglutaryl-CoA lyase (*HMGCL*), lactate dehydrogenase (*LDHA*), mammalian Target of Rapamycin (*mTOR*), v-akt murine thymoma viral oncogene 1 (*AKT1*), NA+/K+ATPase (*ATP1A1*), monocarboxylate transporter, isoform I (*SLC16A1*/MTC1), monocarboxylate transporter, isoform III (*SLC16A3*/MTC4), sodium/proton exchanger, isoform 1 (*SLC9A1*/ NHE-1), sodium/proton exchanger, isoform 2 (*SLC9A2*/NHE-2), sodium/proton exchanger, and isoform 3 (*SLC9A3*/NHE-3) were designed online using the *Primerblast* software (http://www.ncbi.nlm.nih.gov/tools/primer-blast/). Primer sequences for Glyceraldehyde-3-phosphate dehydrogenase (*GAPDH*) and β-actin (*ACTB*) were obtained from [21] and peroxisome proliferator-activated receptor alpha (*PPARA*) and β-hydroxybutyrate dehydrogenase-1 (*BDH1*) from [22, 35] (Table 2).

**Table 2:** Sequence of primers used in RT-PCR reactions, the number of GenBank Accession/EMBL, and the number of base pairs of PCR products.

Gene		Sequence 5’-3’	Accession no. GenBank/ EMBL	Product size, pb
***GAPDH***	F	GTCTTCACTACCATGGAGAAGG	U85042	197
	R	TCATGGATGACCTTGGCCAG		
***ACTB***	F	CAGGATGCAGAAAGAGATCACTGC	NM_173979.3	222
	R	AGGGTGTAACGCAGCTAACAG		
***PPARA***	F	AGGGCTGCAAGGGTTTCTTTAG	NM_001034036	363
	R	TGACGAAAGGCGGGTTGTTGTTG		
***PPARD***	F	CAAAGAGGCTGACGGGAACT	NM_001083636.1	75
	R	CCGGTCATAGCTCTGGCATC		
***BDH1***	F	CCAGGTTATCGCGAACGCAC	NM_001034600.2	103
	R	CAAAAGGCAGAATGGTCAGGC		
***HMGCS1***	F	AGTGAAACTGGCACAGAGCA	NM_001206578.1	133
	R	TCAGGATGTTAGTTCAGGAGGG		
***HMGCL***	F	TACGTCTCCTGTGTGCTTGG	NM_001075132.1	72
	R	CTTGGTGACCTCAGCGACTT		
***LDHA***	F	CATTCATGGGCAGGTAGGACA	NM_174099.2	70
	R	CAGCTGATCCTTGAGAGTTGC		
***mTOR***	F	CCCCAGCTGATTCCACACAT	XM_001788228.1	73
	R	ACTTGATGGAGACGATGCGG		
***AKT1***	R	CTGCACAAGCGAGGTGAGTA	NM_173986.2	134
	F	GAGAAGTTGTTGAGGGGCGA		
***ATP1A1***	F	ATGCCGAAGTGCTGGAATCA	NM_001076798.1	60
	R	TTGGCAGTGATGGGATGGTC		
***SLC16A1***	F	GACGAGCGAACTAGGCAGC	NM_001037319	121
**(MTC1)**	R	CAAGTATCGTTATACGCGGCTC		
***SLC16A3***	F	GGCTGGGATGTACATGTGGT	NM_001109980.1	68
**(MTC4)**	R	ACAAAAGCAGGCAGATCATTTTC		
***SLC9A1***	F	CTGGTGGAAAGTGGAGGCAT	NM_174833.2	196
**(NHE-1)**	R	TGTGTCTGTTGTAGGACCGC		
***SLC9A2***	F	CAGATCCGTCAGCGAACCTT	XM_604493.9	126
**(NHE-2)**	R	GCTGTCCTTCCGAATGCTCT		
***SLC9A3***	F	TCTCCGTGTACAGGGCCATA	AJ131764.1	104
**(NHE-3)**	R	GCCATAGCACATGACCACCT		

EMBL = European Molecular Biology Laboratory; F = Forward; R = Reverse; *GAPDH* = Glyceraldehyde3-phosphate dehydrogenase; *ACTB* = β-actin; *PPARA* = peroxisome proliferator-activated receptor alpha; *PPARD* = peroxisome proliferator-activated receptor delta; *BDH1* = β-hydroxybutyrate dehydrogenase-1; *BDH2* = β-hydroxybutyrate dehydrogenase-2; *HMGCS1* = 3-Hydroxymethylglutaryl-CoA synthase, isoform I; *HMGCL* = 3-Hydroxymethylglutaryl-CoA lyase; *LDHA* = lactate dehydrogenase; *mTOR* = Mammalian Target of Rapamycin; *AKT1* = v-akt murine thymoma viral oncogene 1; *ATP1A1* = NA+/K+ATPase; *SLC16A1* = Monocarboxylate transporter, isoform I (MTC1); *SLC16A3* = Monocarboxylate transporter, isoform III (MTC4); *SLC9A1* = Sodium/proton exchanger, isoform 1 (NHE-1); *SLC9A2* = Sodium/proton exchanger, isoform 2 (NHE-2); *SLC9A3* = Sodium/proton exchanger, isoform 3 (NHE-3).

Real-time PCR analysis were performed using the ABI 7300 real-time PCR System (Thermo Fisher Scientific, Waltham, Massachusetts, USA). For each gene, an optimum amplification condition (cDNA and primer concentration) was selected to achieve a standardized efficiency among all selected genes. For that, five different concentrations of cDNA were used in duplicate. Each reaction was composed of the best cDNA concentration, 0.1 μM of specific primer sets, 7.5 μL power SYBR^®^ green PCR master mix (Thermo Fisher Scientific, Waltham, Massachusetts, USA), and nuclease-free water for a final volume of 15 μL. Amplification conditions consisted of 2 minutes at 50 °C, 10 min at 95 °C, followed by 40 cycles (95 °C for 15 sec and 60 °C for 1 min). After each run of qPCR, the melt curve analysis was performed for each sample to confirm that a single specific product was generated.

Each sample was run in duplicate. The gene expression levels were calculated relative to the average mRNA levels of the internal control gene that was expressed more (*GAPDH* or *ACTB*), according to [12].

### Statistical analysis

Data were analyzed with SAS version 9.0 (SAS Institute Inc., Cary, NC). Weekly averages of weight gain and body measurements were analyzed using a repeated measures mixed model (PROC MIXED), including calf as a random effect and treatment, week, and their interaction as fixed effects. Variables with a single measurement during the study were analyzed by using the GLM procedure, differences among treatments, were analyzed using a Tukey adjustment for *P*-values. Least squares means for each treatment are reported. The variables BW at birth and total serum protein were considered covariates for intake, performance, body frame development, and internal organ weight (% of empty BW). Significance was declared at *P* ≤ 0.05.

Gene expression data were analyzed using SAS version 9.4 (SAS Institute Inc., Cary, NC). It was done via analysis of variance with interaction to delta cycle threshold (ΔCT) averages of *ATPA1*, *BHD1*, *HMGCL*, *HMGCS1*, *LDHA*, *PPARA*, *PPARD*, *SLC16A1* (*MTC1*), *SLC16A3* (*MTC4*), *SLC9A1* (*NHE1*), *SLC9A2* (*NHE2*), *SLC9A3* (*NHE3*), *AKT1*, and *mTOR* genes. The genes were a dependent variable and differences among treatments were assessed using *P*-values.

## Results and discussion

No difference (*P*> 0.05) was observed in the DM intake of the liquid diet among treatments (Table 3, 502.4 ± 13.2 g DM/d), probably because of the restricted volume offered for all treatments.

**Table 3:** Intake, performance, feed efficiency, and body measurements development of calves fed pelleted vs. ground starter with or without hay during the preweaning period.

Item	Treatment			SEM	*P*-value^2^		
	Control (n = 13)	Ground (n = 12)	GH^1^ (n = 13)		T	W	T x W
**Intake**							
**Whole milk (g of DM/d)**	501	503	502	0.70	0.44	0.01	0.99
**Starter (g of DM/d)**	326	314	365	16.7	0.62	0.01	0.99
**Hay (g of DM/d)**	-	-	17	-	-	-	-
**Total DM (g of DM/d)**	827	818	885	17.1	0.42	0.01	0.85
**Total OM (g of DM/d)**	709	711	762	12.9	0.34	0.01	0.78
**Total NDF (g of DM/d)**	135	141	162	7.4	0.54	0.01	0.92
**Water (kg/d)**	2.5	2.9	2.9	0.10	0.72	0.01	0.99
**Performance**							
**Birth BW (kg)**	34.3	33.0	33.9	0.23	0.06	-	-
**Final BW (kg)**	68.7	68.1	71.3	1.35	0.54	-	-
**ADG (g/d)**	541	531	606	18.3	0.25	0.01	0.83
**Feed efficiency**	0.64	0.64	0.67	0.02	0.78	0.01	0.83
**Body measurements (cm)**							
**Withers height**	81.2	81.1	80.6	0.27	0.66	0.01	0.90
**Heart girth**	82.5	82.8	82.0	0.38	0.76	0.01	0.97
**Hip height**	84.7	84.9	84.5	0.27	0.88	0.01	0.71
**Hip width**	22.3	22.5	22.3	0.10	0.73	0.01	0.83

^1^Ground starter + 5% chopped Tifton (*Cynodon* spp.) hay.

^2^Treatment, week and interaction treatment x week.

The physical form of the starter and the dietary inclusion of hay did not influence the intake of nutrients and water (*P*> 0.05; Table 3). However, as in the study by [23], a numerical difference in consumption and weight gain favorable to the GH treatment was observed. Improvements in starter intake and performance when forage was offered to preweaned calves were reported [24], [25]. The positive effects of forage on starter intake may be related to the improvement in rumination, ruminal environment and enhanced muscular development of the rumen [24], [26], and can justify the numerical differences observed. Starter intake (g DM/d) increased linearly as calves grew older (*P*<0.01; Table 3), from 40 ± 33 g DM/d at 1 week old to 722 ± 282 g DM/d at 9 weeks of age.

The intake of hay in the GH treatment was 17 ± 17 g DM/d and increased linearly as calves grew older, with a maximum value of 42 ± 17 g DM/d at 9 weeks of age, corresponded to 4.6% of that offered (forage: starter ratio offered of 5:95).

There was no effect of treatments on body development measurements (*P*> 0.05; Table 3). No differences in body weight of calves receiving hay during preweaning were also reported [23].

Doubling birth weight at 56 d of age is suggested as a weaning criterion [27]. In our study, the initial and final BW were, on average, 33.7 ± 4.4 and 69.4 ± 8.3 kg, respectively. These values were similar between treatments, demonstrating that the diets supplied in all treatments were adequate to reach this criterion.

The apparent digestibility of DM, organic matter (OM), CP, and GE and energy partitioning parameters were similar between treatments (*P*> 0.05; Table 4), showing that the physical form of the starter and inclusion of 5% hay was not able to cause an increase or decrease in the digestibility of the diet. Generally, high fiber diets [28, 29] compromise diet digestibility. However, in the present study this was not observed, possibly due to the restricted inclusion of hay in the diet (5%). Values of 80% of dry matter digestibility were reported for calves receiving different sources of forage [30], corroborating with our results.

**Table 4:** Apparent nutrient digestibility of calves fed control or ground starter with or without hay during the preweaning period.

Item	Treatment			SEM	*P*-value
	Control (n = 6)	Ground (n = 6)	GH^2^ (n = 6)		
**Apparent nutrient digestibility**					
**Dry matter (%)**	83.0	85.4	83.4	0.80	0.45
**Organic matter (%)**	87.2	89.2	87.7	0.47	0.17
**Crude protein (%)**	87.3	88.5	88.5	0.52	0.15
**Gross energy (%)**	86.0	88.5	86.8	0.48	0.50
**Energy partition**					
**Gross energy intake (Mcal/d)**	5.2	5.5	5.8	0.27	0.73
**Fecal energy (Mcal/d)**	0.7	0.6	0.8	0.04	0.47
**Fecal energy (% gross energy intake)**	14.0	11.5	13.2	0.52	0.15
**Digestible energy intake (Mcal/d)**	4.5	4.8	5.0	0.23	0.70
**Urine energy (Mcal/d)**	0.07	0.08	0.07	0.005	0.72
**Urine energy (% gross energy intake)**	1.5	1.6	1.3	0.10	0.44
**Metabolizable energy intake (Mcal/d)**	4.4	4.8	5.0	0.23	0.69
**Metabolizable energy (Mcal/kg DM)**	4.1	4.3	4.2	0.04	0.37
**Metabolizable energy/Gross energy**	1.0	1.0	1.0	0.01	0.46
**Metabolizability (Metabolizable energy/Gross energy)**	0.8	0.8	0.8	0.01	0.20
**Nitrogen balance**					
**N intake (g/d)**	40.0	41.5	43.8	2.39	0.81
**Urine output. kg/d**	12.1	10.1	11.0	0.89	0.67
**N fecal output. g/d**	5.0	4.7	5.1	0.29	0.89
**N Retention (g/d)**	22.8	26.7	27.8	1.71	0.48
**N efficiency**^1^ **(%)**	56.7	63.7	63.7	1.76	0.17

^1^N efficiency % = (N retention/N intake) × 100.

^2^Ground starter + 5% chopped Tifton (*Cynodon* spp.) hay.

Since the different treatments did not yield differences in nutrient digestibility, it is possible to conclude that the inclusion of 5% hay in the diet of calves during the pre weaning does not alter the digestibility of nutrients. This statement is reinforced by the results reported by [31], evaluating crossbred Holstein x Gyr calves fed a complete diet consisting of 6 L/d of liquid diet with 13.5% of total solids and a commercial starter, similar to the one used in the present study, verified digestibilities of 85, 88, 85, and 88% for DM, OM, CP, and GE, respectively, in a trial performed between 50 and 55 d of age.

The energy partitioning parameters were similar between treatments (*P*> 0.05; Table 4). Gross energy intake (Mcal/day) and fecal energy loss (Mcal/d) did not differ between treatments, which led to similar intakes of DE (Mcal/d), GE, metabolizability (ME/GE) (*P*> 0.05), and the parameters relative to the N balance. However, the efficiency of N retention in relation to N ingested in this study was higher to that observed by [32], averaging 40%, in crossbred Holstein x Gyr calves fed 4L/d milk and ground starter. This difference may be related to the different protein content of the diets in the experiments, since increased N intake has a positive correlation with N retention [33].

The similarity in feed efficiency observed between all treatments corroborates with the results observed by [34], who reported that there was no difference in performance, DM intake, nor feed efficiency between calves fed different physical forms of starter (coarse starter with 7.5% bromegrass hay and ground starter with 15% hay). Other studies [35,36] found higher feed efficiency in calves fed pelleted starter compared to other types of processing. However, the dietary inclusion of forage (chopped straw, the average intake of 100 g/d, 72% NDF, 8% CP, and 92% DM) with a textured starter increased feed efficiency concerning the starter without straw [37].

The physical form of starter and the dietary inclusion of 5% hay did not affect ruminal parameters (*P*> 0.05; Table 5), with the exception of N-NH_3_. An interaction between week and N-NH_3_ concentration was observed, with higher N-NH_3_ values at week 8 compared to weeks 4 and 6 for calves fed ground starter. For calves fed Control treatment, there was no difference between the weeks evaluated; for the treatment GH, weeks 4 and 8 were similar, and the lowest values were verified at week 6, which also did not differ from week 4.

**Table 5:** pH, ruminal ammonia nitrogen (N-NH_3_), and organic acids in the rumen of calves fed pelleted vs. ground starter with or without hay during the preweaning period.

Item	Treatment			Week			SEM	*P*-value^2^		
	Control (n = 6)	Ground (n = 6)	GH^1^ (n = 7)	4	6	8		T	W	T x W
**pH**	5.3	5.3	5.3	5.4	5.3	5.3	0.04	0.87	0.54	0.18
**N-NH**_**3**_ **(mg/dL)**	27.6	20.3	19.1	20.6	21.6	24.9	0.95	0.01	0.04	0.02
**Acetate (%)**	41.1	35.0	41.7	35.9	39.0	42.8	1.53	0.36	0.07	0.77
**Propionate (%)**	42.8	38.2	47.9	37.1b	43.3ab	48.6a	1.88	0.28	0.01	0.67
**Butyrate (%)**	5.0	5.0	4.4	5.0	4.9	4.4	0.35	0.81	0.82	0.80
**Acetate/propionate ratio**	1.14	1.01	0.99	1.17a	1.05ab	1.00b	0.05	0.74	0.05	0.13
**Total C2, C3 e C4 (mmol/L)**	132.4	111.6	124.3	114.4b	116.4b	137.5a	4.24	0.15	0.03	0.52

^1^Ground starter + 5% chopped Tifton (*Cynodon* spp.) hay.

^2^Treatment, week and interaction treatment x week.

Molar proportions of acetate, propionate, butyrate and, the sum of three VFA were not different among treatments. An increase in propionate concentrations was observed between the weeks evaluated, with the highest value being observed at week 8, indicating active fermentation at that age. This increase in propionate concentrations led to a reduction in the propionate acetate ratio, and values close to 1.1. This relationship between acetate and propionate was also reported by [38] in the treatment that received a pelleted starter and [39] in the treatment that received a mixture of steam flaked corn and extruded soybeans.

The mean pH 5.3 ± 0.4 was not differing between treatments and weeks. This result suggests that the physical form of starter and the inclusion of 5% hay in the diet with ground starter was not able to increase salivation or the VFA absorption, leading to this similarity between the treatments for pH values. This pH value is associated with ruminal acidosis in adult animals [40]; however, we did not observe signs of bloating, liquid feces nor gas bubbles, which are clinical signs of acidosis. According to [41], calves are apparently able to tolerate lower rumen pH values in comparison to adult animals, which may explain the potential adaptation of ruminal epithelium to starter fermentation [12]. However, subacute ruminal acidosis in young calves has received scant attention, and the severity of rumen acidosis caused by different factors is not yet clear in dairy calves [42].

The empty BW was similar between treatments (*P*> 0.05; Table 6). These results demonstrate that the intake of up to 5% hay did not affect digestive tract fill nor the performance of animals, corroborating with the results of [23]. There were no effects of treatments on reticulorumen, omasum, abomasum, and liver weights (*P*> 0.05; Table 6).

**Table 6:** Weight (% of empty BW) of internal organs and viscera, and gastrointestinal tract development of calves fed pelleted vs. ground starter with or without hay during the preweaning period.

Item	Treatment			SEM	*P*-value
	Control (n = 6)	Ground (n = 6)	GH^1^ (n = 7)		Treatment
**Final BW (kg)**	67.9	67.2	74.4	1.7	0.16
**Empty BW (kg)**	64.5	65.6	71.4	1.85	0.24
**Organ weight (% of empty BW)**					
**Reticulo-rumen**	1.7	1.8	1.8	0.08	0.12
**Omasum**	0.3	0.3	0.3	0.02	0.47
**Abomasum**	0.5	0.6	0.6	0.02	0.18
**Liver**	2.0	2.2	2.1	0.05	0.37
**Height (mm)**					
**Ruminal papillae**	1.49	1.53	1.52	0.03	0.92
**Omasum papillae**	0.70	0.74	0.72	0.03	0.93
**Area (mm**^**2**^**)**					
**Ruminal papillae**	2.07	2.16	1.98	0.09	0.74
**Omasum papillae**	0.98	0.76	0.88	0.07	0.53
**Mitotic index**					
**Rumen**	0.86	0.89	0.74	0.08	0.80
**Omasum**	1.15	1.07	0.76	0.08	0.10

^1^Ground starter + 5% chopped Tifton (*Cynodon* spp.) hay.

Histological parameters of rumen and omasum were not affected by treatments (*P*> 0.05; Table 6). All treatments used the same starter with the same ingredients, differing only in their physical form, since the ground starter was obtained by grinding the Control starter. The absence of differences for all the performance and development parameters showed that the physical form of starter (Control, Ground, or GH) had no effect on the development and performance of calves. These results allow us to hypothesize that the differences frequently pointed out between studies may occur due to the different ingredients and nutritional values of feedstuffs used.

According to [43] the particle size and nutritional factor could affect the expression of genes involved in cellular growth, ion binding, cell proliferation and microbial fermentation. The highest mRNA abundance to monocarboxylate transporter MCT4 was observed on rumen epithelial cells cultured from calves that were stimulated with acetate and propionate [44].

The transporter of the family *NHE* on the cell membrane help maintain the acid-base balance through the exchange of Na^+^ into and H^+^ out of the cell, and it was upregulated by an increase in VFA concentration [45, 46], while the family of the transporters *MTC* is able to transfer of monocarboxylic acid, such as lactic acid, pyruvic acid, and ketones bodies [47, 48].

We did not find difference in mRNA expression level when it was comparing the treatments and the mRNA expression of *ATPA1*, *BHD1*, *HMGCL*, *HMGCS1*, *LDHA*, *PPARA*, *PPARD*, *MTC1*, *MTC4*, *NHE1*, *NHE2*, *NHE3*, *AKT1*, and *mTOR* genes (Table 7). The analysis of gene expression occurred at 65 d of age, and some studies showed that calves were not producing ketones at a rate, similar to that of a mature ruminant until 60 d of age, which is only at 40% of the ketogenic rate of a mature ruminant at 30 d of age [49]. Highest mRNA abundance to monocarboxylate transporter MCT1 and 4, on rumen epithelial cells cultured, was observed in calves during postweaning (55 to 58 d old) than in the preweaning period (22 to 34 d old) [44].

**Table 7:** Effects of the diets with pelleted vs. ground starter with or without hay about mRNA expression level of enzymes *ATPA1*, *BHD1*, *HMGCL*, *HMGCS1*, *LDHA*, transcription factors *PPARA* and *PPARD*, ruminal transporters *MTC1*, *MTC4*, *NHE1*, *NHE2* and *NHE3* and signaling pathway *AKT1* and *mTOR* in the ruminal epithelium.

Genes	Treatment			SEM	*P*-value
	Control (n = 6)	Ground (n = 6)	GH^2^ (n = 7)		Treatment
***ATPA1***	5.42	5.76	5.08	1.70	0.65
***BDH1***	6.26	6.92	6.18	1.54	0.49
***HMGCL***	4.30	4.75	4.00	1.29	0.42
***HMGCS1***	8.45	10.58	8.92	2.53	0.13
***LHDA***	10.01	10.82	10.05	2.14	0.61
***PPARA***	11.48	12.47	11.38	2.80	0.61
***PPARD***	8.32	9.33	8.42	2.04	0.46
***MTC1***	8.62	9.24	8.69	1.65	0.63
***MTC4***	12.70	11.64	10.89	2.40	0.19
***NHE1***	8.30	8.37	8.47	1.87	0.97
***NHE2***	5.69	7.10	5.60	1.76	0.10
***NHE3***	7.86	9.05	7.85	2.24	0.37
***AKT1***	9.93	10.46	9.45	1.79	0.43
***mTOR***	9.39	9.00	9.72	2.42	0.79

^1^Means of the delta cycle threshold (ΔCT) values

^2^Ground starter + 5% chopped Tifton (*Cynodon* spp.) hay.

## Conclusions

The physical form of starter evaluated in this study (pellet with steam-flaked corn, ground, or ground + 5% chopped Tifton hay) did not influence performance, feed efficiency, nutrient partitioning, nor rumen development of dairy calves. We did not find a difference in mRNA expression of genes involved in rumen metabolism. In this way, starter pellet with steam-flaked corn, ground, or ground + 5% chopped Tifton hay can be used for suckling calves.

## Supporting information

S1 Data (XLSX)
